# Evaluation of temocillin efficacy against KPC-2-producing *Klebsiella pneumoniae* isolates in a hollow-fibre infection model

**DOI:** 10.1093/jac/dkae027

**Published:** 2024-02-09

**Authors:** José Luis Rodríguez-Ochoa, Patricia Pérez-Palacios, Vicente Merino-Bohórquez, Miriam Ortiz-Padilla, Ana Velázquez-Escudero, Jesús Rodríguez-Baño, José Manuel Rodríguez-Martínez, Álvaro Pascual, Fernando Docobo-Pérez

**Affiliations:** Unidad Clínica de Enfermedades Infecciosas y Microbiología, Hospital Universitario Virgen Macarena, Sevilla, Spain; Unidad Clínica de Enfermedades Infecciosas y Microbiología, Hospital Universitario Virgen Macarena, Sevilla, Spain; Instituto de Biomedicina de Sevilla IBIS, Hospital Universitario Virgen Macarena/CSIC/Universidad de Sevilla, Sevilla, Spain; Unidad de Gestión de Farmacia Hospitalaria, Hospital Universitario Virgen Macarena, Sevilla, Spain; Departamento de Farmacología, Facultad de Farmacia, Universidad de Sevilla, Sevilla, Spain; Unidad Clínica de Enfermedades Infecciosas y Microbiología, Hospital Universitario Virgen Macarena, Sevilla, Spain; Instituto de Biomedicina de Sevilla IBIS, Hospital Universitario Virgen Macarena/CSIC/Universidad de Sevilla, Sevilla, Spain; Unidad Clínica de Enfermedades Infecciosas y Microbiología, Hospital Universitario Virgen Macarena, Sevilla, Spain; Unidad Clínica de Enfermedades Infecciosas y Microbiología, Hospital Universitario Virgen Macarena, Sevilla, Spain; Departamento de Medicina, Facultad de Medicina, Universidad de Sevilla, Sevilla, Spain; Centro de Investigación Biomédica en Red en Enfermedades Infecciosas (CIBERINFEC), Instituto de Salud Carlos III, Madrid, Spain; Instituto de Biomedicina de Sevilla IBIS, Hospital Universitario Virgen Macarena/CSIC/Universidad de Sevilla, Sevilla, Spain; Centro de Investigación Biomédica en Red en Enfermedades Infecciosas (CIBERINFEC), Instituto de Salud Carlos III, Madrid, Spain; Departamento de Microbiología, Facultad de Medicina, Universidad de Sevilla, Avda. Sánchez Pizjuán s/n., 41009 Sevilla, Spain; Unidad Clínica de Enfermedades Infecciosas y Microbiología, Hospital Universitario Virgen Macarena, Sevilla, Spain; Instituto de Biomedicina de Sevilla IBIS, Hospital Universitario Virgen Macarena/CSIC/Universidad de Sevilla, Sevilla, Spain; Centro de Investigación Biomédica en Red en Enfermedades Infecciosas (CIBERINFEC), Instituto de Salud Carlos III, Madrid, Spain; Departamento de Microbiología, Facultad de Medicina, Universidad de Sevilla, Avda. Sánchez Pizjuán s/n., 41009 Sevilla, Spain; Instituto de Biomedicina de Sevilla IBIS, Hospital Universitario Virgen Macarena/CSIC/Universidad de Sevilla, Sevilla, Spain; Centro de Investigación Biomédica en Red en Enfermedades Infecciosas (CIBERINFEC), Instituto de Salud Carlos III, Madrid, Spain; Departamento de Microbiología, Facultad de Medicina, Universidad de Sevilla, Avda. Sánchez Pizjuán s/n., 41009 Sevilla, Spain

## Abstract

**Background:**

Temocillin is an old antimicrobial that is resistant to hydrolysis by ESBLs but has variable activity against carbapenemase-producing Enterobacteriaceae. The current EUCAST susceptibility breakpoints for Enterobacterales are set at ≤16 mg/L (susceptible with increased exposure) based on a dose of 2 g q8h, but there is limited information on the efficacy of this dose against temocillin-susceptible carbapenemase-producing *Klebsiella pneumoniae* isolates.

**Objectives:**

To evaluate the efficacy of this dose using a hollow-fibre infection model (HFIM) against six KPC-2-producing clinical isolates of *K. pneumoniae*.

**Methods:**

The isolates were characterized by WGS and temocillin susceptibility was determined using standard and high inoculum temocillin. Mutant frequencies were estimated and temocillin activity was tested in time–kill assays and in the HFIM. At standard conditions, three of the isolates were classified as susceptible (MIC ≤ 16 mg/L) and three as resistant (MIC > 16 mg/L). The HFIM was performed over 3 days to mimic human-like pharmacokinetics of 2 g q8h. Bacterial counts were performed by plating on Mueller–Hinton agar (MHA) and MHA containing 64 mg/L temocillin to detect resistant subpopulations.

**Results:**

All isolates showed a reduction in bacterial population of at least 3 log cfu/mL within the first 8 h of simulated treatment in the hollow-fibre assay. Regrowth was observed for the three resistant isolates and one of the susceptible ones. The MIC value for these isolates was higher by at least two dilutions compared with their initial values.

**Conclusions:**

These data suggest that an optimized pharmacokinetic regimen may be of clinical interest for the treatment of KPC-2-producing *K. pneumoniae* susceptible to temocillin. These data showed activity of temocillin against KPC-2-producing *K. pneumoniae* susceptible to temocillin; however, a dose of 2g q8h administered over 30 min may be inadequate to prevent the emergence of resistant variants.

## Introduction

The number of ESBL-producing Enterobacteriaceae has increased exponentially over recent decades.^1^ Carbapenems have been considered the last therapeutic option for invasive infections caused by these microorganisms, as they are not hydrolysed by ESBL or AmpC-type β-lactamases.^2^ However, the main consequence of their use has been an increase in the emergence of carbapenem-resistant Enterobacteriaceae (CRE).^3^ As a result, since 2017, the WHO has classified them as ‘priority 1’ critical pathogens in the search for new therapeutic alternatives or the rescue of old molecules.^4^

Temocillin, a semi-synthetic 6-α-methoxy derivative of ticarcillin, was developed in the 1980s and is currently approved in the UK, the Netherlands, France and Germany for the treatment of urinary tract infections, respiratory tract infections and bacteraemia.^5^ This methoxy group has been shown to confer resistance to class A and C β-lactamases by preventing the entry of a water molecule into the active centre of the enzyme.^5^ However, one of the main reasons for its lack of widespread use is its narrow spectrum of activity, due to its weak interaction with PBPs 1, 3 and 4 and its lack of activity against *Pseudomonas aeruginosa*, *Acinetobacter baumannii* or Gram-positive bacteria.^6–10^

The current temocillin EUCAST breakpoints for Enterobacterales are set for *Escherichia coli*, *Klebsiella* spp. (excluding *Klebsiella aerogenes*) and *Proteus mirabilis* only, based on two different doses: 2 g every 12 h as standard dose for the treatment of susceptible isolates (MIC < 0.001 mg/L) and 2 g every 8 h as a high-exposure dosing regimen (MIC ≤ 16 mg/L).^11^ Recently, temocillin pharmacokinetics were investigated in plasma and epithelial lining fluid (ELF) of infected neutropenic mice and the plasma exposure–response relationships were determined for ESBL-producing *E. coli* and *Klebsiella pneumoniae* isolates.^12^ Temocillin activity against KPC-2-producing clinical isolates of *E. coli* was also satisfactorily evaluated in a murine peritoneal sepsis model.^13^ In view of its efficacy results, it would be worthwhile to evaluate its activity against carbapenemase-producing *K. pneumoniae* isolates. Therefore, the aim of this study was to determine the efficacy of the increased dose of temocillin against KPC-2-producing *K. pneumoniae* isolates, both susceptible and resistant to temocillin, in an *in vitro* dynamic hollow-fibre model.

## Materials and methods

### Bacterial isolates

Six carbapenemase-producing *K. pneumoniae* clinical isolates (KP#181, KP#182, KP#191, KP#192, KP#201, KP#202) were selected from the Reference Laboratory for the Surveillance and Control of Nosocomial Infections and Prudent Use of Antimicrobials Program in Andalucía (PIRASOA program, Hospital Universitario Virgen Macarena, Seville, Spain) because of their susceptibility to temocillin. Molecular typing and characterization of the antimicrobial resistance determinants of the clinical isolates and those derived from the hollow-fibre infection model (HFIM) experiment were performed by WGS using the MiSeq system (Illumina, San Diego, CA, USA). The DNA sample library was prepared using the Nextera XT DNA library preparation kit (Illumina). Raw reads were quality filtered and assembled *de novo* using CLC Genomic Workbench v10 (QIAGEN). ST was assigned using MLSTFinder databases (https://cge.food.dtu.dk/services/MLST/) and the annotation of the antimicrobial resistance determinants was carried out using ResFinder 4.4.1 (http://genepi.food.dtu.dk/resfinder).^14^

### In vitro susceptibility studies

Temocillin susceptibility testing was performed following EUCAST guidelines using broth microdilution according to ISO 20776-1 in microtitre plates.^11^ Temocillin (BOC Sciences, USA) concentrations ranged from 0 to 512 mg/L and *E. coli* ATCC 25922 was used as the control strain. The assays were conducted in triplicate in Mueller–Hinton broth (MHB). Susceptibility categories were assigned using EUCAST v13.1 clinical breakpoints.^11^ Additionally, the effect of higher bacterial concentration on the temocillin susceptibility was performed using the broth microdilution method with bacterial concentrations of 5 × 10^7^ and 5 × 10^8^ cfu/mL.

### Temocillin-resistant mutant frequency studies

The frequency of emergence of temocillin-resistant mutants was evaluated for the six selected clinical isolates. Briefly, an overnight culture of each strain (10^9^ cfu/mL) was diluted 1:10^6^ to avoid the presence of mutants in the initial culture. An initial inoculum of 10^3^ cfu/mL was incubated overnight in MHB and subsequently placed on drug-free plates (to calculate the total bacterial concentration) and on Mueller–Hinton agar (MHA) plates containing 32 mg/L temocillin. Mutants were isolated from MHA plates supplemented with temocillin at concentrations of 4× MIC of each isolate and 32 mg/L (corresponding to the first concentration to be considered as temocillin resistant). Plates were incubated for 24 h at 37°C. The experiments were performed in triplicate.

### Temocillin activity in static assays

The temocillin activity was evaluated in time–kill assays using a microdilution method according to CLSI guidelines.^15^ An initial inoculum of 1 × 10^6^ cfu/mL was prepared and plated on 96-well plates containing concentrations of temocillin ranging from 0.25 to 512 mg/L. A growth control (no temocillin) and a ‘no growth’ control (no inoculum) were used. The plates were incubated for 18 h at 37°C. cfu/mL counts were determined at 0, 2, 4, 8 and 24 h by plating properly diluted samples onto drug-free MHA plates. The assays were performed in duplicate and the lower limit of detection was set at 2 log_10_. A bactericidal effect was defined as a ≥3 log_10_ (99.9% killing) decrease in cfu at the time specified.^15^

### Temocillin activity in dynamic infection model

Additionally, the temocillin activity was assessed in an HFIM with human-like temocillin pharmacokinetic profiles. An initial bacterial burden of 1 × 10^6^ cfu/mL was used for the HFIM.

The activity of temocillin against the six carbapenemase-producing clinical isolates of *K. pneumoniae* was assessed in a dynamic *in vitro* HFIM using polyethersulfone hollow-fibre cartridges (Aquamax HF03, Nikkiso, Belgium) in MHB. The unbound temocillin concentration and time profiles were adjusted to mimic those observed in human plasma after IV administration of 2 g q8h.^16^ Based on *in silico* simulations in ADAPT,^17^ antimicrobial concentration–time profiles were performed in the HFIM by adjusting flow rates to replicate target concentrations and half-lives (*t*_½_ of 5 h).^16^ The extracapillary space of each HFIM was inoculated with 50 mL of bacterial suspension using an inoculum of 10^6^ cfu/mL and incubated at 37°C. Bacterial concentrations were determined at 0, 1, 2, 4, 6, 8, 24, 48 and 72 h. Serial dilutions were then plated on both drug-free and drug-containing MHA plates (temocillin 64 mg/L, 4-fold the breakpoint concentration for susceptibility) to count total and resistant bacterial populations, respectively.

The assays were performed in duplicate and the lower limit of detection was 2.0 log_10_ cfu/mL. Plates were incubated for 18 h at 37°C for subsequent counting of cfu/mL. For the sake of simplicity, the definition of bactericidal activity used for the time–kill assays was used for the HFIM.

### Drug assay for pharmacokinetics

The temocillin concentration was determined within the first dosing interval and at steady state. One millilitre was drawn from the central compartment on Days 1 and 2 at selected timepoints (0, 1, 2, 4, 6, 8, 24 h). Samples were stored immediately at −80°C until analysis.

Temocillin concentrations were measured using a liquid chromatograph associated with a diode array UV spectrophotometer (HPLC-DAD, Agilent 1260 Infinity, Waldbronn, Germany) as described by Miranda *et al.*^18^ with modifications. The validated working concentration range was linear from 1 to 100 µg/mL (R^2 ^> 0.9998), the accuracy was within 96%–101% and the coefficients of variation in intraday and interday precision were less than 10% and within the precision range of 95%–105%. The lower limit of quantification (LLOQ) was 1 µg/mL.

## Results

### Bacterial isolates, in vitro susceptibility studies and mutant frequency

Isolates KP#192, KP#201 and KP#202 were susceptible to temocillin with MIC values of 4, 2 and 8 mg/L, respectively. The MIC for KP#181 and KP#182 isolates was 64 mg/L, and 32 mg/L for KP#191, these three being resistant to temocillin according to the EUCAST breakpoints (Table 1). KP#181 and KP#182, and KP#202 belonged to the high-risk clones ST307 and ST15, respectively. The data derived from the WGS have been deposited in the ENA database (BioProject ID: PRJEB69516). No mutants were detected at 4× MIC for KP#181, KP#182, KP#191 and KP#202, but frequencies of >10^8^ were observed for KP#192 and KP#201. At temocillin concentrations of 32 mg/L no mutants emerged with KP#192, KP#201 or KP#202.

**Table 1.** Characteristics, susceptibility and mutant frequency of KPC-2-producing *K. pneumoniae* isolates

Isolates	β-Lactam resistance genes	ST	Temocillin MIC (mg/L)		Mutant frequency	
			Standard conditions	High bacterial burden	4× MIC	Susceptibility breakpoint of 32 mg/L
KP#181	*bla*_KPC-2_, *bla*_TEM-1_, *bla*_CTX-M-15_, *bla*_OXA-1_	307	64	>1024	<1.43 × 10^−9^	ND
KP#182	*bla*_KPC-2_, *bla*_TEM-1_, *bla*_OXA-1_	307	64	>1024	<1.66 × 10^−9^	ND
KP#191	*bla*_KPC-2_, *bla*_TEM-1_	1562	32	>1024	<1.69 × 10^−9^	ND
KP#192	*bla*_KPC-2_, *bla*_TEM-1_	37	4	>1024	1.50 × 10^−8^	<1.73 × 10^−9^
KP#201	*bla_KPC-2_*, *bla_TEM-1_*, *bla_SHV-52_*	485	2	>1024	3.86 × 10^−6^	<3.67 × 10^−9^
KP#202	*bla_KPC-2_*, *bla_TEM-1_*, *bla_SHV-28_*	15	8	>1024	<1.51 × 10^−9^	<1.51 × 10^−9^

ND, not done.

### Temocillin time–kill assays

**Figure 1.**
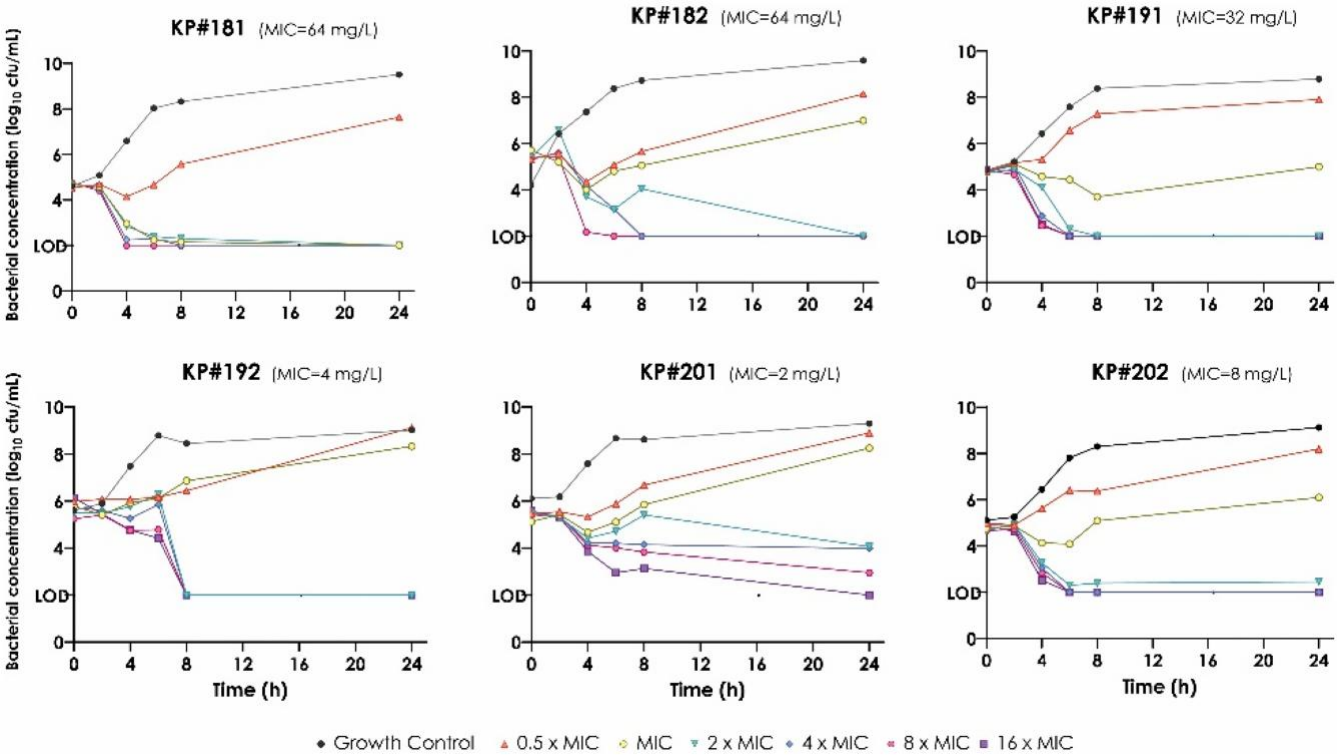
Temocillin time–kill assay over 24 h at concentrations ranging from 0 to 512 mg/L. This figure appears in colour in the online version of *JAC* and in black and white in the print version of *JAC*.

The results of the time–kill curves are shown in Figure 1. All the isolates regrew at temocillin concentrations below or equivalent to their respective MICs. However, total bacterial eradication or at least no bacterial regrowth at concentrations above their MICs were observed. Bactericidal activity was observed at 2× MIC in all isolates except isolate KP#201 (16× MIC).

An initial bacterial decrease was observed for the temocillin-resistant strains KP#181 at 0.5× MIC (32 mg/L) and for KP#182 at 0.5× MIC (32 mg/L) and 1× MIC (64 mg/L). KP#191 showed the same behaviour at concentrations of 1× MIC (32 mg/L) and 2× MIC (64 mg/L). However, bacterial regrowth occurred after 8 h.

Regarding susceptible strains, the KP#202 isolate showed similar behaviour, with a 1 log_10_ cfu/mL decrease within the first 6 h of temocillin exposure, with a subsequent regrowth. With strain KP #192, a sudden decline was observed at concentrations of 2× MIC (8 mg/L) or greater, showing from bacterial counts in the range of 4–6 log_10_ at 6 h to negative cultures after 8 h.

### HFIM

#### Temocillin pharmacokinetics

**Figure 2.**
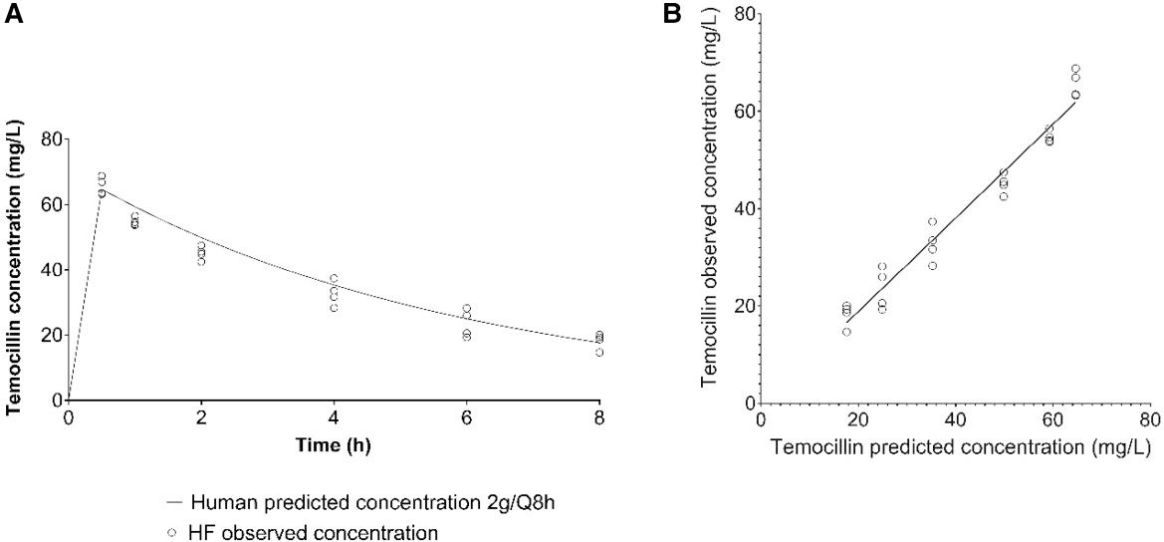
Pharmacokinetic profiles in the HFIM after dosages of temocillin 2 g q8h. (a) The solid line shows the predicted concentrations and open circles the observed concentrations. (b) Scatterplot showing observed versus predicted antimicrobial concentrations. The open circles and the solid line represent individual observed-predicted data points and the linear regression of observed/predicted values, respectively.

The observed versus predicted concentrations of temocillin in the HFIM after temocillin (2 g q8h) dosages are shown in Figure 2. The correlation between the predicted and observed temocillin pharmacokinetic profiles in HFIM is shown in Figure 2 [R^2^ = 0.96; intercept, 17.939 (95% CI, 7.14–28.78); slope, 1.06 (95% CI, 0.889–0.99)].

#### Effect of temocillin on total bacterial population

**Figure 3.**
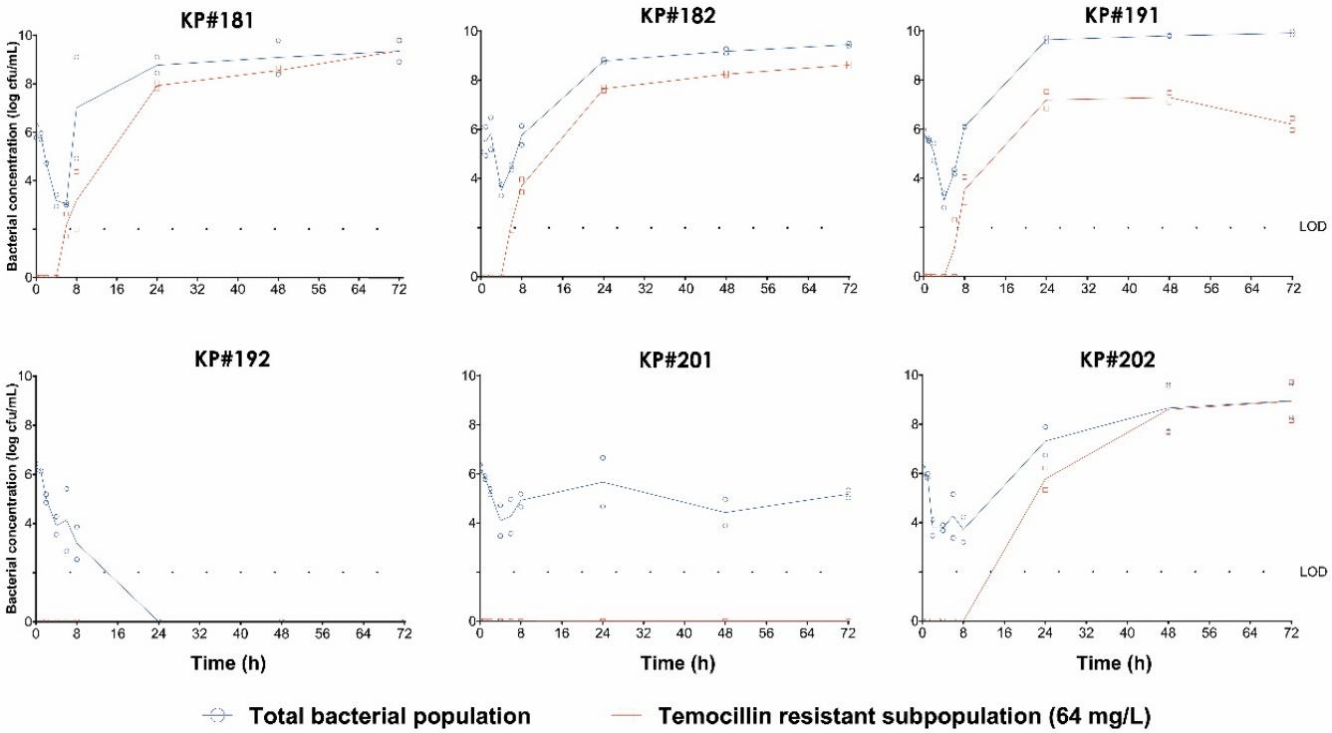
HFIM of KPC-2-producing *K. pneumoniae* mimicking a temocillin dosage of 2 g q8h. Open circle and square symbols denote total and temocillin-resistant bacterial populations, respectively. The limit of detection (LOD) was set at 2 log_10_ cfu/mL. This figure appears in colour in the online version of *JAC* and in black and white in the print version of *JAC*.

The temocillin-resistant isolates (KP#181, KP#182, KP#191) showed a bacterial reduction of 3 log_10_ cfu/mL within the first 8 h (Figure 3). After that, the total population kept growing until maximum population (10 log_10_ cfu/mL) at 24 h. Regarding the susceptible isolates, two of the three strains (KP#201 and KP#202) showed a bacterial reduction of 2 log_10_ within the first 8 h. KP#202 was able to regrow, reaching a maximum bacterial density at 48 h. KP#201, after an initial 2 log_10_ reduction, maintained a stationary population in the range of 4–6 log_10_ cfu/mL during the entire assay (similar to the initial inoculum). Only KP#192 was eradicated, showing a bacterial reduction of 2 log_10_ after 8 h and achieving negative cultures at 24 h.

#### Effect of temocillin on emergence of resistance

Resistant subpopulations, defined as those that were able to grow in medium with a concentration of 64 mg/L temocillin, were observed with the KP#181, KP#182, KP#191 and KP#202 isolates, emerging 6 h after the start of the assay (Figure 3). The maximum bacterial population was reached at 24 h in the case of KP#181, KP#182 and KP#191. With respect to KP#202, resistant variants emerged at 8 h and the maximum population was achieved after 48 h. In contrast, KP#192 and KP#201 did not show resistant subpopulations. At the end of the assays the strains KP#181, KP#191 and KP#202 increased their MICs of temocillin to >1024, 128 and >1024 mg/L, respectively.

With respect to the mutations found in the genomes of KP#201 and KP#202 after the HFIM, we were unable to detect any difference in KP#201 between the original isolate and the one obtained at the end of the assay. On the other hand, KP#202 showed a mutation [V295G (GTC → GGC)] in the sensor histidine kinase (BaeS) of the two-component system BaeS-BaeR and a mutation [Q321R (CAG → CGG)] in the subunit MdtC of the multidrug efflux pump RND permease.

## Discussion

To the best of our knowledge, there are no studies investigating the efficacy of temocillin using human-like temocillin exposures against carbapenemase-producing *K. pneumoniae* isolates. The present study investigated the activity of a human-like temocillin exposure after administration of 2 g q8h against six KPC-2-producing clinical isolates of *K. pneumoniae* (three resistant and three susceptible). There are two key findings from this study. First, although this posology did not eradicate resistant isolates, an initial reduction of 3 log_10_ cfu/mL was observed. Secondly, isolates classified as susceptible showed a different behaviour.

To observe the effect of this dose on resistant strains, isolates with MIC values 4-fold (isolates KP#181 and KP#182) and 2-fold (KP#191) the susceptibility breakpoint value for temocillin were selected. All isolates were able to grow again after Day 3, but the growth curves showed two distinct trends throughout the treatment period. Initially, a reduction in the bacterial population of up to 3 log_10_ was observed, reaching a minimum after approximately 8 h of treatment. This could be explained by the simulated temocillin concentrations, which reached a *C*_max_ (64 mg/L) 30 min after the start of the assay and decreased to 16 mg/L after 8 h. This initial bacterial decrease is consistent with the time–kill assays, in which a concentration of 64 mg/L, corresponding to the *C*_max_ observed in critically ill patients,^16^ was bactericidal in all isolates. On the other hand, bacterial regrowth was observed after 8 h due to the emergence of resistant subpopulations in all resistant isolates and in one susceptible isolate.

The frequency of occurrence of temocillin-resistant mutants for the isolates showing this regrowth was less than 10^−9^, which is in agreement with previous studies.^19^ All resistant isolates and one susceptible isolate showed the emergence of subpopulations with elevated MIC. However, these results cannot be explained by the pre-existence of temocillin-resistant subpopulations because the initial bacterial concentration was lower than the mutant frequency observed for each of the isolates. However, this parameter was assayed in a single step and the temocillin concentrations tested were higher than those simulated in the HFIM. Temocillin resistance has been described to occur when there is sequential exposure to this drug,^5^ which may be more likely under the conditions simulated in the HFIM.

Isolate KP#202 was the susceptible isolate that was able to regrow after the HFIM assay. This could be explained by the detected mutations [V295G (GTC → GGC)] in the sensor histidine kinase (BaeS) of the two-component system BaeS-BaeR, as shown by Guerin *et al*.,^20^ and the second mutation [Q321R (CAG → CGG)] in the subunit MdtC of the multidrug efflux pump RND permease. Also, we could not rule out the possibility that the effect of the temocillin inoculum may also have played an important role in this isolate. Our results were in agreement with those of Adams *et al*.,^19^ who described a mild inoculum effect for temocillin at a concentration of 6 log_10_ cfu/mL (equal to the initial inoculum in this infection model) in *K. pneumoniae* isolates expressing KPC-2, resulting in a 2-fold increase in MIC.

For the isolate KP#201, although no resistant subpopulation was observed, complete eradication of the inoculum was not achieved. The total bacterial population remained stable throughout the assay, as did its MIC. It is important to note that in the time–kill assays KP#201, unlike the other isolates, showed that it was necessary to increase its MIC 16-fold to achieve bactericidal effect. In this sense we cannot rule out the possibility of tolerance phenotype as previously described for other β-lactams.^21,22^

This study has some limitations as the HFIM lacks the effect of the host immune response, which could enhance bacterial killing against both susceptible and resistant isolates. In addition, temocillin is typically administered through continuous infusion in the ICU. Nevertheless, the present study used a fractionated dose using pharmacokinetic data derived from the ICU patient population. However, we aimed to use an antimicrobial exposure similar to that used by EUCAST for designing susceptibility breakpoints for the susceptible increased exposure category (2 g q8h). Moreover, the effect of inter-individual pharmacokinetic variability on bacterial killing and resistance emergence could not be fully characterized. Finally, six clinical *K. pneumoniae* isolates were tested and the results may not apply to other *K. pneumoniae* isolates, other carbapenemases or infections with other Gram-negative bacillary species.

In conclusion, exposure to 2 g q8h of temocillin achieved extensive initial bacterial killing *in vitro* against KPC-2-producing *K. pneumoniae*. However, this exposure did not prevent the emergence of highly resistant temocillin subpopulations in temocillin-resistant isolates in a susceptible isolate. An optimized pharmacokinetic regimen or the addition of a second antimicrobial agent may be of clinical interest for the treatment of KPC-2-producing *K. pneumoniae* susceptible to temocillin.
